# Simvastatin protects auditory hair cells from gentamicin-induced toxicity and activates Akt signaling *in vitro*

**DOI:** 10.1186/1471-2202-12-114

**Published:** 2011-11-14

**Authors:** Yves Brand, Cristian Setz, Soledad Levano, Alwin Listyo, Eduardo Chavez, Kwang Pak, Michael Sung, Vesna Radojevic, Allen F Ryan, Daniel Bodmer

**Affiliations:** 1 Department of Biomedicine, University Hospital Basel, Hebelstrasse 20, 4031 Basel, Switzerland; 2 Clinic of Otolaryngology, Head and Neck Surgery, University Hospital Basel, Petersgraben 4, 4031 Basel, Switzerland; 3 San Diego VA Medical Center, 3350 La Jolla Village Drive, La Jolla, CA 92037, USA; 4 Surgery/Otolaryngology, UCSD School of Medicine, 9500 Gilman Drive MC0666, La Jolla, CA 92093-0666, USA; 5 Neurosciences Departments, UCSD School of Medicine, 9500 Gilman Drive MC0666, La Jolla, CA 92093-0666, USA

## Abstract

**Background:**

Inhibitors of 3-hydroxy-3-methylglutaryl-coenzyme A reductase, known as statins, are commonly used as cholesterol-lowering drugs. During the past decade, evidence has emerged that statins also have neuroprotective effects. Research in the retina has shown that simvastatin, a commonly used statin, increases Akt phosphorylation *in vivo*, indicating that the PI3K/Akt pathway contributes to the protective effects achieved. While research about neuroprotective effects have been conducted in several systems, the effects of statins on the inner ear are largely unknown.

**Results:**

We evaluated whether the 3-hydroxy-3-methylglutaryl-coenzyme A reductase is present within the rat cochlea and whether simvastatin is able to protect auditory hair cells from gentamicin-induced apoptotic cell death in a *in vitro *mouse model. Furthermore, we evaluated whether simvastatin increases Akt phosphorylation in the organ of Corti. We detected 3-hydroxy-3-methylglutaryl-coenzyme A reductase mRNA in organ of Corti, spiral ganglion, and stria vascularis by reverse transcriptase-polymerase chain reaction (RT-PCR). Moreover, we observed a dose-dependent and significant reduction of hair cell loss in organs of Corti treated with simvastatin in addition to gentamicin, as compared to samples treated with gentamicin alone. The protective effect of simvastatin was reversed by addition of mevalonate, a downstream metabolite blocked by simvastatin, demonstrating the specificity of protection. Finally, Western blotting showed an increase in organ of Corti Akt phosphorylation after simvastatin treatment *in vitro*.

**Conclusion:**

These results suggest a neuroprotective effect of statins in the inner ear, mediated by reduced 3-hydroxy-3-methylglutaryl-coenzyme A reductase metabolism and Akt activation.

## Background

Until recently, sensorineural hearing loss due to damage to cochlear hair cells (HC) has been regarded as an inevitable consequence of age, genetic conditions or exposure to certain environmental stimuli. During the past several years, some of the critical intracellular events that mediate damage to HCs have been discovered, using aminoglycoside-induced HC death *in vitro *as a model [1-4]. It has been demonstrated that small GTPases, such as Ras and Rho/Rac/Cdc42, as well as the c-Jun-N-terminal kinase signalling pathway, are activated in cells exposed to the drug and that phoshatidylinositol-3-kinase (PI3K) signalling mediates HC survival and opposes gentamicin toxicity via its downstream target, the protein kinase AKT [5-9]. After prolonged aminoglycoside exposure, caspases are activated and HCs undergo apoptotic cell death [10,11].

Inhibitors of 3-hydroxy-3-methylglutaryl-coenzyme A (HMG-CoA) reductase, otherwise known as statins, are commonly used as cholesterol-lowering drugs. Statins reduce the incidence of primary and secondary coronary heart disease in clinic trials and act by blocking the enzyme necessary for the production of L-mevalonate, an intermediary product in the synthesis of cholesterol [12,13]. During the past decade, evidence has emerged that statins also have neuroprotective effects. Animal models suggest that statins may be beneficial in the treatment of multiple sclerosis and during acute stroke [14-20]. Several *in vitro *and *in vivo *studies provided evidence that statins activate the protein kinase B (PKB/Akt) pathway [21,22]. Work done in the retina has shown that simvastatin, a commonly used statin, increases Akt phosphorylation *in vivo*, indicating that the PI3K/Akt pathway contributes to central nervous system protective effects achieved [23].

In the inner ear, Cai et al. found that simvastatin protected the hearing of mice deficient in apolipoprotein E that were fed a high fat diet [24]. However, they attributed this effect to control of hyperlipedemia. Syka et al. demonstrated that atrovastin slows down the deterioration of inner ear function with age in mice. They suggested that atrovastin reduces endothelial inflammatory effects that influence the blood supply to the inner ear [25]. While no experiments were performed, Borghi et al. hypothesized that statins might be useful as a treatment for sensorineural hearing loss due to their metabolic and hemodynamic effects [26]. However, a prospective, randomized, double-blinded clinical trial by Olzowy et al. did not show an effect of atrovastin on progression of sensorineural hearing loss in the elderly [27]. In contrast, Chiu et al. reported that simvastatin exposure produced damage to lateral line HCs in the zebrafish, although the mechanism was not identified [28].

Given these conflicting data, we determine whether or not HMG-CoA reductase is present within the rat cochlea, and whether simvastatin is able to protect mammalian auditory HCs from gentamicin-induced HC death. Given the results of Chiu et al. we also evaluated simvastatin for HC toxicity [28]. In addition we investigated the metabolic pathway involved in simvastatin effects, and whether this drug increases Akt phorphorylation in the organ of Corti (OC).

## Results

### HMG-CoA reductase mRNA is expressed in the cochlea

**Figure 1:**
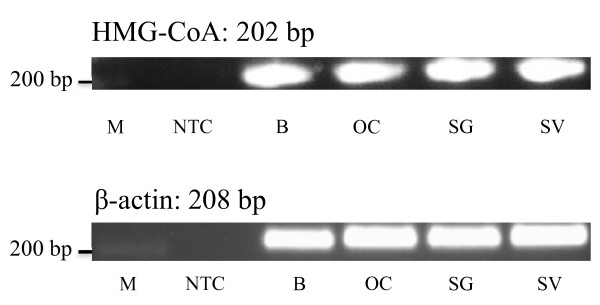
**Detection of HMG-CoA reductase in the cochlea of 5-day-old Wistar rats**. Total RNA of the organ of Corti (OC), the spiral ganglion (SG) and the stria vascularis (SV) was reversed transcribed into cDNA and amplified by PCR. Total brain (B) RNA served as a positive control. Omission of cDNA in the PCR mixture served as no template control (NTC). The DNA ladder (M) is indicated with its lengths. β-actin was used to control cDNA synthesis.

HMG-CoA mRNA were detected in the OC, spiral ganglion (SG), and stria vascularis (SV) using specific primer sets (Table 1). The amplification of β-actin confirmed a successful synthesis of cDNA. The specificity of the designed primers was confirmed using cDNA from rat brain tissue. One single band of the correct size for every tissue was observed (Figure 1). Omission of cDNA in the PCR mixture served as negative control.

**Table 1:** Primer sequences used for HMG-CoA reductase and β-actin.

Gene	Primer name	Sequence 5' → 3'	Annealing temperature	Exons	Product length
HMG-CoA reductase	Forward	TGTTCAAGGGGCGTGCAAAGACAA	63	17	202 bp
				
	Reverse	TCAAGCTGCCTTCTTGGTGCATGT		18	

β-actin	Forward	ACGGTCAGGTCATCACTATCGGCA	58	3	208 bp
				
	Reverse	ATCCTGTCAGCAATGCCTGGGT		4	

### Simvastatin has no toxic effect on HCs and protects HCs from gentamicin-induced HC damage in vitro

**Figure 2:**
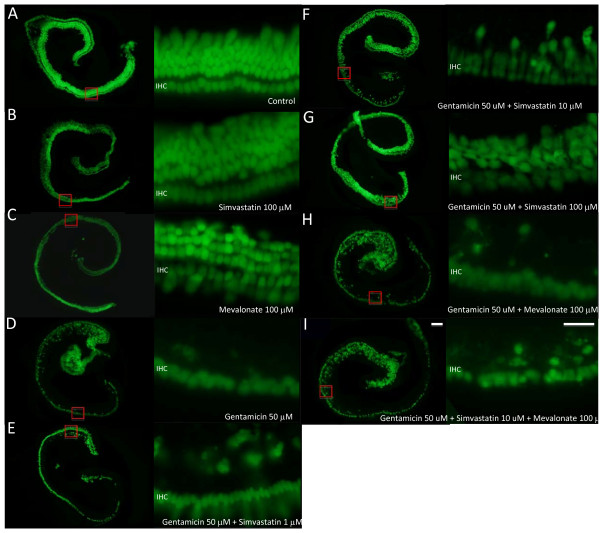
**Effect of simvastatin on gentamicin-induced hair cell damage**. Photograph of organs of Corti (OCs). Overview on the left (scale bar 100 μm) and high magnification on the right (sale bar 20 μm). Untreated OCs (**A**), OCs exposed to 100 μM simvastatin (**B**), and OCs exposed to 100 μM mevalonate demonstrate three orderly rows of outer hair cells (OHC) and a single row of inner hair cells (IHC). OCs cultured with gentamicin showed significant loss of hair cells (**C**). Treatment with increasing concentrations of simvastatin in addition to gentamicin resulted in dose dependent significant decrease in hair cell loss compared with gentamicin treatment only (**D-F**). Mevalonate reverses the protective effect of simvastatin on gentamicin-induced hair cell damage. OCs cultured with mevalonate in addition to gentamicin and OCs cultured with simvastatin 10 μM and mevalonate showed the same degree of hair cell loss as OCs cultured with gentamicin only (**H,I**).

To exclude a toxic effect of simvastatin, the OCs were cultured with the highest dosages used in this study (100 μM) for 72 hours. The number of HCs was compared between cultured OCs in presence and absence of simvastatin. Because no difference was found, a toxic effect of simvastatin was excluded. Untreated control OCs and those treated with simvastatin showed three orderly rows of outer hair cells (OHCs) and a single row of inner hair cells (IHCs) (Figure 2).

**Figure 3:**
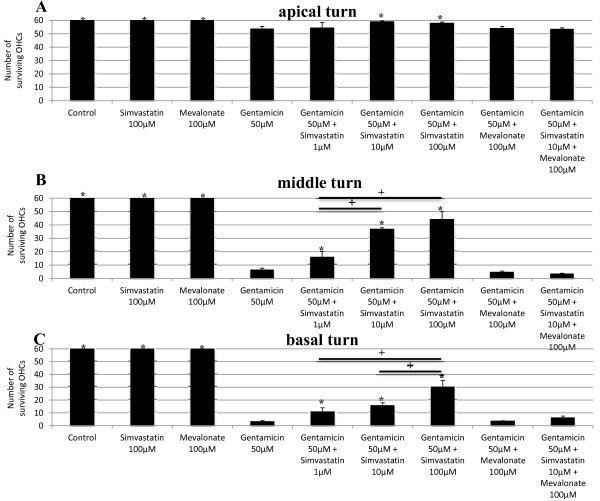
**Quantitative analysis of surviving outer hair cells (OHCs) by cochlear turns**. Data are expressed as the mean number of surviving OHCs per 20 IHCs. Vertical lines represent SEM. **A**) Average OHC survival in the apical turn in the different experimental conditions. There was a statistically significant increase in OHC survival between the groups treated with simvastatin in the concentration of 10 μM and 100 μM in addition to gentamicin compared with gentamicin treatment only (p < 0.05). * indicates significant difference from gentamicin treatment only. **B**) Average OHC survival in the middle turn in the different experimental conditions. There was a statistically significant increase in OHC survival between the groups treated with simvastatin in addition to gentamicin compared with gentamicin treatment only (p < 0.05 simvastatin 1 μM, p < 0.01 simvastatin 10 μM and simvastatin 100 μM). The protective effect of simvastatin was dose dependent. * indicates significant difference from gentamicin treatment only. + indicates significant difference between the indicated groups (p < 0.01).**C**) Average OHC survival in the basal turn in the different experimental conditions. There was a statistically significant increase in OHC survival between the groups treated with simvastatin in addition to gentamicin compared with gentamicin treatment only (p < 0.05 simvastatin 1 μM, p < 0.01 simvastatin 10 μM and simvastatin 100 μM). The protective effect of simvastatin was dose dependent. * indicates significant difference from gentamicin treatment only. + indicates significant difference between the indicated groups (p < 0.01).

As expected, gentamicin treatment led to a loss of HCs (Figure 2). Treatment with both gentamicin and simvastatin at the concentration of 1 μM significantly increased OHC survival in the middle and basal cochlear turn (Figure 3). Whereas treatment with both gentamicin and simvastatin at the concentration of 10 μM and 100 μM, resulted in increased OHC survival in all cochlear turns (Figure 3). The protective effect of simvastatin on gentamicin-induced HC damage was dose dependent in the middle and basal turn (Figure 3).

### Mevalonate does not effect gentamicin-induced HC damage in vitro and has no toxic effect on hair cells in vitro itself

To exclude a toxic effect of mevalonate, the OCs were cultured with the dosages used in this study (100 μM) for 72 hours. The number of HCs was compared between cultured OC in presence and absence of mevalonate. Because no difference was found, a toxic effect of mevalonate was excluded. Untreated control OCs and those treated with mevalonate alone showed three orderly rows of OHCs and a single row of IHCs (Figure 2). Treatment with gentamicin and mevalonate showed no statistically significant difference in OHC survival compared to treatment with gentamicin only in all cochlear turns (Figure 3).

### Mevalonate reverses the protective effect of simvastatin on gentamicin-induced HC damage in vitro

Treatment with simvastatin 10 μM in addition to gentamicin showed significantly less OHC loss in all cochlear turns than groups treated with gentamicin only or with simvastain 10 μM and mevalonate 100 μM in addition to gentamicin (Figure 3). The protective effect of simvastatin was reversed by the addition of mevalonate, a downstream metabolite. This bypasses the effect of simvastatin on the upstream enzyme, 3-hydroxy-3-methylglutaryl-coenzyme A, demonstrating the mechanism of protection.

### Simvastatin increases Akt phosphorylation in vitro

**Figure 4:**
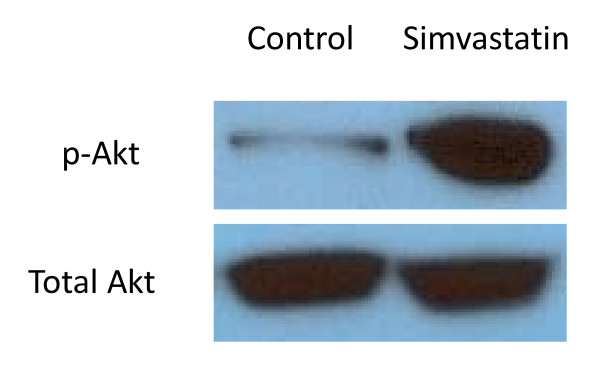
**Representative Western blots of phosphorylated Akt and total Akt**. Organs of Corti were either exposed for 1 hour to control media (Control) or media containing 10 μM simvastatin (Simvastatin).

Western blotting revealed specific activation of Akt in OC treated with simvastatin *in vitro *(Figure 4). Blots using anti-pAkt revealed a strong increase in activated Akt, after a 1 hour exposure to 10 μM simvastatin. p-Akt has been referenced to total Akt in the control and simvastatin treated group (Figure 4).

## Discussion

At present, little is known about the effects of statins on the inner ear. It has been hypothesized that statins might be useful as a treatment for sudden sensorineural hearing loss due to their metabolic and hemodynamic effects [26]. Syka et al. demonstrated that atrovastin had a positive effect on the deterioration of inner ear function with age in mice by analysing distortion product otoacoustic emissions (DPOAE). Treated mice showed decreased expression of intracellular and vascular adhesion molecules in the aortic wall and the authors suggest that reduced endothelial inflammatory effects may contribute to better OHC survival by influencing blood supply to the inner ear [25]. While Syka et al. attribute the positive effect of statins on inner ear function on hemodynamic effects, other reports suggest that statins act through their metabolic effects. It has been reported that simvastatin may prevent hearing loss and inner ear damage in apolipoprotein E gene knockout mice fed a high-fat diet, by reducing atherosclerotic lesions and levels of glucose, cholesterol, low density lipoproteins, and triglyceride [24]. Therefore it was suggested that statins might be used to treat hearing loss associated with hyperlipedemia.

Toxic effects of simvastatin on the inner ear have also been reported. Park et al. found that simvastatin treatment induced morphological alterations and apoptosis in murine cochlear neuronal cells [29]. Chiu et al. reported that simvastatin was toxic to lateral line HCs in the zebrafish [28]. Given these conflicting data, we evaluated the effects of simvastatin on cochlear HCs *in vitro*. We demonstrate that HMG-CoA reductase mRNA is expressed in the cochlea. We excluded a toxic effect of simvastatin exposure on auditory HCs. Treatment with simvastatin in addition to gentamicin led to significant decrease in HC loss compared with the gentamicin group. Although simvastatin enhanced HC survival, it did not provide complete protection against gentamicin-induced HC loss. Western blotting reveals that simvastatin increased Akt phosphorylation in the OC *in vitro*, indicating that the PI3K/Akt pathway may contribute to the protective effects achieved. It is significant that the protective effect of simvastatin was reversed by addition of mevalonate, since this finding demonstrates the specificity of protection to the metabolic pathway that is regulated by statins.

It should be noted that in our experiments cochlear explants were harvested from postnatal day 3-5 animals since only newborn animals can be used for extended culture of inner ear HCs. Adult HCs do not survive in culture. In a large number of studies, aminoglycosides have been utilized as inducers of HC death and the immature cochlea is an established *in vitro *model. However, younger animals are more sensitive to ototoxins [3,4,7] and therefore our results must generalize to adults with caution. It should be noted that for the RT-PCR and Western blotting experiments we used Wistar rat pups, whereas for the *in vitro *experiment transgenic mouse pups in which expression of green fluorescent protein (GFP) is driven by an auditory HC specific promoter were used. The rationale for this is that the larger cochlea of the rat allows more tissue to be harvested and fewer animals needed to be sacrificed for the experiments. The transgenic mouse pups were chosen for the *in vitro *experiments in order to easily visualize the GFP-positive HCs, and to monitor HC loss while the cells were alive.

The possibility that simvastatin might interact physically with gentamicin must also be considered. Although we have no direct evidence excluding physical interaction, the fact that the protective effect of simvastatin on gentamicin-induced HC loss was reversed by adding mevalonate argues against it.

How can the protective effect of simvastatin in gentamicin-induced HC loss be explained? To date there are no reports of simvastatin-induced intracellular events on HCs available. Certain evidence suggests that the reduction of cholesterol cannot entirely account for statins' neuroprotective effects and it has been demonstrated that short-term statin treatment does not alter cholesterol level in the brain. This indicates that statins have another mechanism of action, possibly through the other products of the mevalonate pathway that play a role in cellular signalling [30,31]. This is not surprising since mevalonate is not only essential for the biosynthesis of cholesterol, but also for other products, such as Coenzyme Q10 (Q10) and isoprenylated proteins, which are essential in several cell processes [22,32].

Several *in vitro *and *in vivo *studies provided evidence that statins activate the PKB/Akt pathway [21,22]. Work done in the retina has shown that simvastatin increases Akt phosphorylation *in vivo*, indicating that the PI3K/Akt pathway contributes to the central nervous system protective effects achieved [26]. In a previous study, we demonstrated that PI3K mediates HC survival and opposes gentamicin toxicity in neonatal rat OC [9]. Therefore, activation of Akt by simvastatin as demonstrated here is one explanation for the protective effects of the drug on gentamicin-induced HC loss. A study on mammalian endothelial cells demonstrated that simvastatin activates Akt in these cells, while treatment with mevalonate blocked this activation of Akt [22]. This finding directly links HMGCoA reductase -mevalonate metabolism to Akt activation. Our study is the first demonstration, to our knowledge, that this pathway is involved in Akt activation in the inner ear.

**Figure 5:**
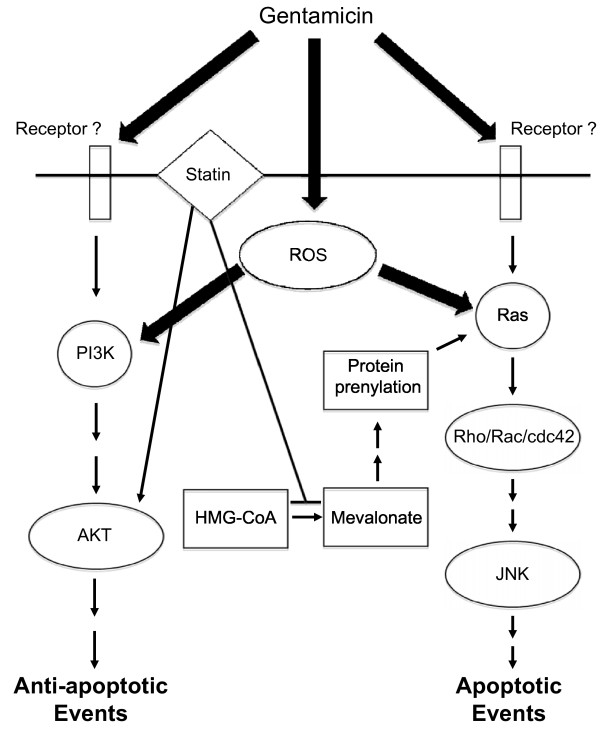
**A simplified model of signal transduction in gentamicin-induced hair cell damage and the mechanisms of statin-mediated protection**. The mode of initial interaction between the HC and gentamicin is not known. It may include a receptor, and/or entry of gentamicin into the cell followed by generation of reactive oxygen species (ROS). We propose that statins act by enhancing Akt activiation and decrease the isoprenylation of small G proteins, such as Ras and Rho/Rac/Cdc42.

A second potential mechanism through which statins can affect cells is by blocking the isoprenylation of small G proteins, such as Ras and Rho/Rac/Cdc42. It has been shown that statins downregulate the activity of small G proteins in cardiomyocytes in culture and *in vivo *[33]. In previous studies, we showed that inhibition of the small GTPases Rho/Rac/Cdc42 or specific blocking of Ras provided potent protection against gentamicin-induced auditory HC loss [5,6]. Since mevalonate alone does not affect HC death, this implies that sufficient prenylated Ras is normally present in HCs, and that additional mevalonate does not enhance damage signaling. However, we did not evaluate the role of small G proteins in this study. Our conclusions on the protective effect of simvastatin in gentamicin-induced HC loss are summarized in Figure 5.

As mentioned above, simvastatin has been shown to have a toxic effect on fish lateral line HCs and cochlear neurons [28,29]. The concentration of simvastatin used in this study is in the same range used on cochlear neurons [29]. Although this prior work did not address mammalian HCs, potential toxic effects need to be considered. Mevalonate is essential for the production of coenzyme Q10 and statins lead to a dose-dependent reduction in Q10 [34]. Q10 is an important mitochondrial antioxidant that helps bypass existing mitochondrial respiratory chain defects. Mitochondrial predispositions and dysfunction are thought to play a major role in adverse effects of statins, and it has been demonstrated that water-soluble Q10 promotes OHC survival in a guinea pig model of noise induced hearing loss [35,36]. We hypothesize that zebrafish HCs and mouse cochlear neurons might be more vulnerable to Q10 reduction by simvastatin than mouse cochlear HCs. While this hypothesis is perhaps too complex to be attractive without additional supporting data, it is at least consistent with our observations and with the current literature.

## Conclusion

Our results demonstrate a partial protection of OHCs in the OC against gentamicin ototoxicity in vitro. This neuroprotective effect of statins on mammalian auditory HCs is mediated by reduced 3-hydroxy-3-methylglutaryl-coenzyme A reductase metabolism and Akt activation.

## Methods

### Animal procedures

The animal procedures for the RT-PCR were carried out in Basel, Switzerland according to an approved animal research protocol (Kantonales Veterinäramt, Basel, Switzerland) in accordance with the European Communities Council Directive of 24 November 1986 (86/609/EEC). All other animal procedures were carried out in San Diego, CA USA. The local animal subject committee of the VA San Diego Healthcare System approved the animal procedures in accordance with the guidelines laid down by the National Institute of Health regarding the care and use of animals for experimental procedures.

### Tissue extraction

For RT-PCR and western blotting tissue was extracted from 5-day-old Wistar rat pups (Harlan, Indianapolis, IN, USA). For all other experiments 3-day-old transgenic mice pups in which expression GFP is driven by an auditory HC specific *Brn-3.1 *promoter were used [37]. The animals were decapitated, and cochlear microdissections were performed under a light microscope to isolate the OC, the SG, and the SV [38]. Brain tissue was removed from the same animals as a positive control for the RT-PCR. During the microdissection, the different tissues were maintained in ice-cold PBS.

### Tissue culture

For experiments in which cultures of OCs were needed, OCs were first incubated in culture medium [Dulbecco's Modified Eagle Medium supplemented with 10% FCS, 25 mM HEPES and 30 U/ml penicillin (Invitrogen, Carlsbad, CA, USA) at 37°C in 5% CO_2_] and left for 24 hours at 37°C in 5% CO_2 _for recovery. After that period, the OCs were transferred into a new solution and incubated for 48 hours at 37°C in 5% CO_2_. To induce HC damage, OCs were cultured with 50 μM gentamicin (Sigma-Aldrich, St. Louis, MO, USA) in cell culture medium for 48 hours. OCs were pretreated for 24 hours with increasing amounts of simvastatin (Sigma-Aldrich) at the final concentration of 1 μM, 10 μM or 100 μM in the cell culture medium, mevalonate 100 μM in cell culture medium and with simvastatin 10 μM in combination with mevalonate 100 μM in cell culture medium during the 24 hour recovery period after dissection. Before use simvastatin was converted into the active acid following the protocol of Bogman et al. [39]. Stock solutions of 10 μM simvastatin in DSMO were stored at - 20°C. After this pretreatment, OCs were exposed either to gentamicin and simvastatin for 48 hours or to gentamicin in combination with simvastatin and mevalonate for 48 hours. Other OCs were either held in culture medium alone (control), treated with simvastatin at a final concentration of 100 μM or with mevalonate at a final concentration of 100 μM.

### RNA extraction

For PCR, 20 OCs, SGs, SVs and 20-40 mg brain of 5-day-old WS rat pups were separately placed in RNAlater (Qiagen, Hombrechtikon, Switzerland). RNA isolation of brain and inner ear components were performed using the RNAeasy Minikit (Qiagen) including DNase treatment according to the supplier's instructions. To homogenize the tissues, we used homogenizer Ultra-Turrax T8 (IKA-Werke, Staufen, Germany). The quantity and quality of the isolated RNA was determined with NanoDrop ND 1000 (NanoDrop Technologies, Delaware, USA). The 260/280 nm ratio of all our samples was between 1.8 and 2.1.

### Primer design

Gene sequences from HMG-CoA reductase (NM_013134.2) and β-actin (NM_031144.2) were accessed from GenBank. Primers for RT-PCR were designed using Primer-Blast software available at the NCBI (National Center for Biotechnology Information). Our criteria for primer design included *T*_m _values between 58°C and 60°C, a minimum length of 20 nt, a product size of 100-500 bp, with an absence of long G-C stretches. Primers were designed to cross at least one exon junction for the specific amplification of cDNA and to avoid amplification of genomic DNA. The details of primers employed along with the annealing temperatures and product sizes are provided in Table 1.

### Reverse Transcriptase-Polymerase Chain Reaction

Total RNA (1 μg) was reverse transcribed into cDNA with the first-strand cDNA synthesis kit (Roche Applied Biosciences, Rotkreuz, Switzerland) according to the supplier's instructions. PCR was performed using the PCR Master Mix (Roche Applied Biosciences) with primers specific for HMG-CoA reductase. β-actin primers were used as a positive control for cDNA synthesis. The primer sets are described in Table 1. PCR reactions were run in the Eppendorf Mastercycler (Eppendorf, Hamburg, Germany) under the following conditions. For HMG-CoA reductase, we started with an initial denaturation of 94°C for 3 minutes and followed by 30 cycles. Each cycle consisted of denaturation at 94°C for 30 seconds, annealing at 63°C for 30 seconds and extension at 72°C for 30 seconds, with a final extension step at 72°C for 5 minutes. For β-actin, we started with an initial denaturation of 94°C for 3 minutes and followed by 30 cycles. Each cycle consisted of denaturation at 94°C for 30 seconds, annealing at 58°C for 45 seconds and extension at 72°C for 30 seconds, with a final extension step at 72°C for 1 minute. The PCR products were stained with SybrGreen I (Molecular Probes, Oregon, USA), separated by electrophoresis on a 2% agarose gel and visualized under UV light. Omission of cDNA in the PCR mixture served as negative control.

### Hair cell count

OCs were fixed in 4% paraformaldehyde. After fixation, the OCs were visualized and photographed using a fluorescence microscope (Olympus FSX100). Quantitative analysis was obtained by evaluating 60 OHCs associated with 20 IHCs in a given microscope field. Explants were analyzed separately for the apical, middle and basal turn. For each turn, two random microscope fields were counted and averaged. These values were averaged across the six replications of each experiment. Since there was almost no damage to the IHCs (< 5%), only the OHCs were counted and used to analyze HC survival.

Results obtained in the HC counting were analyzed by using analysis of variance (ANOVA) followed by the least significant difference (LSD) post-hoc test (Stat View 5.0). Differences associated with P-values of less than 0.05 were considered to be statistically significant. All data are presented as mean ± SD.

### Assessment of Signaling Protein Activation

To assess the activation of the PIK3/Akt signaling pathway, per condition 6 intact OCs from 5-day-old Wistar rat pups (Harlan) were harvested and placed in cell culture media for 24 hours as described above. They were then placed in cell culture media, with or without 10 μg/ml simvastatin for 1 hour. Explants were collected from media, and lysed with 100 μl T-Per Tissue Protein Extraction Reagent (Thermo Scientific, Rockford, IL, USA) in 1X phosphatase/proteases inhibitors (Roche, Indianapolis, IN, USA) and sonicated for 10 min to shear chromosomal DNA. Samples where centrifuge at 10,000G for 10 minutes to separate cytosolic part from membranous components. Equal quantities of these lysates were separated by Bis-Tris Mini Gels 4-12% gels, and electrotransferred to polyvinylidene difluoride membranes (Bio-Rad, Hercules, CA, USA). The membranes were blocked with 5.5% nonfat dried milk in TBS-Tween [50 mM Tris-HCL (pH 7.4), 150 mM NaCl, 0.05% Tween 20] for 60 min at room temperature. Blots were incubated with primary antibodies in blocking buffer overnight at 4°C and then incubated with horseradish peroxidase-linked secondary antibodies (Jackson Immuno, West Grove, PA, USA) followed by chemiluminescent detection (GE Healthcare, Piscataway, NJ, USA). Blots were evaluated with antibodies against the phosphorylated forms of Akt, and total Akt (both Cell Signaling Technology, Beverly, MA, USA) on the same membrane. Western blotting was replicated three times with independent biological replicas.

## Authors' contributions

YB participated in the design of the study, carried out the RT-PCR, hair cell count and drafted the manuscript. CS, AL, KP, MS and VR participated in the tissue culture for the hair cell count and RT-PCR. SL was involved in the primer design for the RT-PCR and participated in the design of the study. EC carried out the assessment of signaling protein activation by Western blotting. AFR and DB conceived the study, and participated in its design and coordinated and helped to draft the manuscript. All authors read and approved the final manuscript.
